# Preface

**DOI:** 10.2174/157340313805076331

**Published:** 2013-02

**Authors:** Jianyi Zhang

Current Cardiology Reviews (CCR) has continued to grow from last eight years of publication. It is a matter of great prestige that CCR features high quality articles by researchers from all over the world. As an Editor in Chief, I feel accomplished that I am able to contribute in such a reputable Journal which is circulated globally. The interactions with authors have been a worthwhile experience.

I am pleased that manuscripts published in CCR are diverse in the subjects they offer. We have highlighted a range of key areas like congestive heart failure, cardiomyopathy, congenital heart disease, drugs, methodology, pacing, and preventive cardiology. We plan to publish a greater number of papers/articles in years to come which will feature basic science reports in every aspect of cardiovascular science

I take this opportunity to acknowledge the contributions made by referees who participated in peer review process of CCR. We have been fortunate enough to rely on scientists who enjoy expertise in dealing with technical issues in laboratories and check the quality of submitted articles. I am obliged and grateful to the reviewers from different countries who provided their valuable and timely comments to the authors. Often there are delays observed in the publication procedure of the articles and it is somewhat frustrating for authors to wait for extended time periods. We affirm that for all future volumes, we will publish artciles within the given time frame therefore minimizing cause to any inconvenience for authors. In addition, I am thankful to the staff of Bentham Science Publishers for their efforts for more than eight years now; especially to Ms. Sadia Rafique who has contributed and helped in managing the Journal with great efficiency.

